# Short-term effectiveness of low dose liraglutide in combination with metformin versus high dose liraglutide alone in treatment of obese PCOS: randomized trial

**DOI:** 10.1186/s12902-017-0155-9

**Published:** 2017-01-31

**Authors:** Mojca Jensterle, Nika Aleksandra Kravos, Katja Goričar, Andrej Janez

**Affiliations:** 1 Department of Endocrinology, Diabetes and Metabolic Diseases, University Medical Centre Ljubljana, Zaloška 7, Ljubljana, SI-1000 Slovenia; 2 Institute of Biochemistry, Faculty of Medicine, University of Ljubljana, Vrazov trg 2, Ljubljana, 1000 Slovenia

**Keywords:** Liraglutide, Metformin, Obesity, PCOS

## Abstract

**Background:**

Liraglutide 3 mg was recently approved as an anti-obesity drug. Metformin is weight neutral, yet it could enhance the therapeutic index of GLP-1 agonist. We compared weight-lowering potential of liraglutide 1.2 mg in combination with metformin to liraglutide 3 mg monotherapy in obese PCOS.

**Methods:**

Thirty obese women with PCOS (aged 33.1 ± 6.1 years, BMI 38.3 ± 5.4 kg/m^2^) were randomized to combination (COMBO) of metformin (MET) 1000 mg BID and liraglutide 1.2 mg QD (*N =* 15) or liraglutide 3 mg (LIRA3) QD alone (*N =* 15) for 12 weeks. The primary outcome was change in anthropometric measures of obesity.

**Results:**

Both treatments led to significant weight loss (−3.6 ± 2.5 kg, *p =* 0.002 in COMBO vs −6.3 ± 3.7 kg, *p =* 0.001 in LIRA3). BMI and waist circumference reduction in LIRA3 was greater than in COMBO (−2.2 ± 1.3 vs −1.3 ± 0.9 kg/m^2^_,_ *p =* 0.05 and −4.2 ± 3.4 vs −2.2 ± 6.2 cm, *p =* 0.014, respectively). Both interventions resulted in a significant decrease of post-OGTT glucose levels. COMBO significantly reduced total testosterone and was associated with less nausea.

**Conclusions:**

Short-term interventions with COMBO and LIRA3 both led to significant improvement of measures of obesity in obese PCOS, LIRA3 being superior to COMBO. However, COMBO further improved androgen profile beyond weight reduction and was associated with better tolerability.

**Trial registration:**

The study was retrospectively registered with ClinicalTrials.gov (NCT02909933) on 16^th^ of September 2016.

## Background

Obesity is present in 50–80% of women with polycystic ovary syndrome (PCOS) [1]. Even a modest weight loss of 5–10% is primarily important for reduction of cardiovascular risk factors and improvement of fertility potential [2–4]. First line recommended lifestyle therapy for weight management often remains unsatisfactory and non-sustainable in clinical practice [5–7].

Liraglutide, a long acting glucagon-like peptide-1 (GLP-1) analogue, has a dose dependent dual beneficial effect. It improves glucose homoeostasis and reduces body weight. As anti-diabetic therapy is approved at doses up to 1.8 mg [8], whereas higher doses are required for maximum weight reduction [9, 10]. Liraglutide 3 mg led to decreases in body weight of more than 5 to as much as 15% [11] and was recently approved for weight management in many countries. Exposure to higher doses was not linked with deterioration in safety when compared with lower doses but to potential higher frequency of gastrointestinal side effects [12].

Metformin is an established treatment in PCOS. Despite being weight neutral as monotherapy, it has multiple favourable effects mediated through reduction of insulin resistance and via direct action on steroidogenesis. It improves clinical, metabolic and reproductive outcomes of the syndrome [13–17]. In addition, metformin has a less defined and less studied potential to enhance the therapeutic index of GLP-1 [18–22]. We hypothesized that metformin may enhance weight-lowering capacity of low dose liraglutide and provide certain advantages in management of PCOS related obesity compared to high dose liraglutide alone.

The purpose of this pilot randomized study was to compare the combination of metformin and low dose liraglutide 1.2 mg to high dose liraglutide 3 mg alone on measures of obesity in obese PCOS.

## Methods

### Study design

The present study used a 12-week pilot prospective randomized open-label design recruiting 30 obese women with PCOS. It was conducted at the outpatients Department for Endocrinology, Diabetes and Metabolic Diseases University Medical Center Ljubljana. The study was registered according to the Slovenian drug Law and retrospectively registered with ClinicalTrials.gov (NCT02909933) on 16^th^ of September 2016.

The patient inclusion criteria were as follows: type A phenotype of PCOS diagnosed by ASRM-ESHRE Rotterdam criteria including concomitant presence of a) hyperandrogenemia on either the biochemical or the clinical level, b) menses abnormalities and c) PCO morphology; age 18 years to menopause and obesity (body mass index: BMI ≥ 30). Patients with history of carcinoma, significant cardiovascular, kidney or hepatic disease and the use of medications known to affect reproductive or metabolic functions within prior to study entry were excluded.

They were randomized to COMBO or LIRA3 arm. In COMBO arm, metformin was initiated at a dose of 500 mg once per day and increased up to 1000 mg BID. Liraglutide was initiated at a dose of 0.6 mg injected s.c. once per day and increased to 1.2 mg. In LIRA3 arm, liraglutide was initiated at a dose of 0.6 mg injected s.c. once per day with increments to 3 mg. The primary outcome of the study was mean change in measures of obesity. Secondary outcomes included metabolic and hormonal changes.

At baseline and end of the study all patients underwent standard anthropometric measurements: height, weight and waist circumference. BMI was calculated as the weight in kilograms divided by square of height in meters. Patients were provided with glucose-monitoring devices and supplies and educated on their use. They were instructed to measure blood glucose levels at any signs and symptoms suggesting low blood glucose. Hypoglycemia was defined according to American Diabetes Association criteria as symptoms suggestive of low blood glucose confirmed by self-monitored blood glucose measurement below 3.9 mmol/l [23]. All women were instructed to report any side effects during the treatment. They were given an advice on recommended lifestyle intervention that was actively promoted at the beginning of the study.

A fasting blood was drawn for determination of glucose, insulin, androstenedione, total and free testosterone (T) followed by a standard 75 g oral glucose tolerance test (OGTT) to assess glucose homeostasis. Glucose levels were determined using a standard glucose oxidase method (Beckman Coulter Glucose Analyzer, Beckman Coulter Inc CA, USA). Insulin was determined by solid-phase enzyme-labeled chemiluminiscent immunometric assay (Immulite 2000 XPi System, Siemens Healthcare, United Kingdom). Androstenedione was measured by specific double antibody RIA using 125 I-labeled hormones (Diagnostic Systems Laboratories, Webster, Tx). Total and free testosterone levels were measured by coated tube RIA (DiaSorin, S. p. A, Salluggia, Italy and Diagnostic Products Corporation, LA, respectively). Sex hormone binding globulin (SHBG) was determined with a chemiluminescent immunoassay (Immulite 2000 Analyzer, Siemens Healthcare, Erlangen, Germany). Lipids were determined using Adiva 1800, Siemens analyzer. Intra-assay variations ranged from 1.6 to 6.3%, and inter-assay variations ranged from 5.8 to 9.6% for the applied methods. Pre- and post-treatment samples from each patient were assayed in the same assay run. Homeostasis model assessment (HOMA-IR) calculation was applied as a measure for insulin resistance (IR). Safety clinical assessment was performed at the beginning and week 4, 8 and 12 of the treatment period. Pregnancy was excluded before randomization by measuring β-human chorionic gonadotropin. Women were advised to strictly use barrier contraception. They were informed that there is a very limited knowledge based on some case reports regarding human exposure to liraglutide during pregnancy in women with diabetes [24].

### Statistical analysis

Sample size was determined based on mean change in weight and data from previous studies with comparative treatment intervention using Power and Sample Size Calculation version 3.0.43 [25]. To detect a statistically significant difference between groups of approximately 2.5 kg in weight loss with 80% power, each group had to consist of 14 patients. Mean values and standard deviation were used to describe continuous variables. Nonparametric Wilcoxon signed-rank test for related samples was used to compare pretreatment and post-treatment values of continuous variables in each of the treatment groups. To compare the pretreatment values and the change of clinical parameters among different treatment groups, nonparametric Mann–Whitney test was used. Logistic regression was used to compare the proportion of patients losing at least 5% of weight. *P* values of <0.05 were considered statistically significant. All statistical analyses were performed using IBM SPSS Statistics version 19.0 (IBM Corporation, Armonk, NY, USA).

## Results

### Baseline results

The study enrolled 30 participants (aged 33.1 ± 6.1 years, BMI 38.3 ± 5.4 kg/m^2^, mean ± SD). Two patients discontinued the study because of protocol violation. Twenty-eight patients completed the study: 14 on COMBO and 14 on LIRA3. Baseline characteristics of the patients are provided in Table 1. There were no statistically significant differences between both groups (Table 1).

**Table 1:** Pretreatment and posttreatment values of clinical parameters of the patients for each treatment group

	LIRA3	*N =* 14		LIRA1.2 + MET2000	*N =* 14		Comparison of pretreatment values between groups
Characteristic	Pretreatment	Post treatment	*P* ^a^	Pretreatment	Post treatment	*P* ^a^	*P* ^b^
Age (years)	34.6 ± 6.1			31.6 ± 5.9			0.164
Weight (kg)	111.1 ± 14.8	104.7 ± 14.8	**0.001**	102.5 ± 9	98.9 ± 10.3	**0.002**	0.137
BMI (kg/m^2^)	39.2 ± 5.5	37.0 ± 5.5	**0.001**	37.5 ± 5.3	36.2 ± 5.5	**0.002**	0.482
Waist circumference (cm)	110.1 ± 12	105.9 ± 12.8	**0.003**	105.2 ± 10.7	103.0 ± 8.2	0.113	0.265
Androstenedione (nmol/l)	7.0 ± 3.7	7.7 ± 3.4	0.451	10.6 ± 6.4	8.6 ± 3.5	0.530	0.094
Total testosterone (nmol/l)	1.3 ± 0.7	1.2 ± 0.5	0.065	1.8 ± 0.9	1.5 ± 0.8	**0.023**	0.125
Free testosterone (pmol/l)	9.1 ± 5.1	9.7 ± 3.8	0.594	11.7 ± 5.7	11.0 ± 4.2	0.660	0.164
SHBG (nmol/l)	39.6 ± 41.4	46.9 ± 54.5	**0.018**	27.7 ± 21.1	47.2 ± 90.4	0.430	0.137
Glu 0 min OGTT (mmol/l)	5.2 ± 0.4	5.1 ± 0.4	0.341	5.4 ± 0.6	5.0 ± 0.3	**0.015**	0.635
Glu 120 min OGTT (mmol/l)	5.9 ± 1.0	4.7 ± 0.7	**0.002**	7.1 ± 2.0	5.9 ± 1.6	**0.016**	0.178
Insulin 0 min OGTT (mU/l)	22 ± 11.7	19.4 ± 10.4	0.272	24.5 ± 18.4	16.8 ± 13.2	**0.035**	0.839
Insulin 120 min OGTT (mU/l)	86.4 ± 40.3 [2]	68.9 ± 58.4	**0.008**	118.8 ± 87.5	109.1 ± 127.0	0.551	0.631
HOMA IR	5.1 ± 2.9	4.4 ± 2.4	0.272	5.9 ± 4.3	3.7 ± 3.0	**0.013**	0.910
Cholesterol (mmol/l)	4.8 ± 0.8	4.8 ± 0.8	0.875	4.9 ± 1.1 [1]	4.5 ± 1.0 [1]	0.107	0.720
HDL cholesterol (mmol/l)	1.3 ± 0.3	1.2 ± 0.3	0.194	1.3 ± 0.1 [1]	1.3 ± 0.2 [1]	0.627	0.239
LDL cholesterol (mmol/l)	2.9 ± 0.8	3.0 ± 0.7	0.928	3.0 ± 0.9 [1]	2.7 ± 0.9 [1]	**0.049**	0.905
TG (mmol/l)	1.5 ± 0.7	1.6 ± 0.6	0.727	1.4 ± 0.7 [1]	1.2 ± 0.5 [1]	0.075	0.550

data are expressed as mean ± SD

^a^compared with Wilcoxon signed-rank test for related samples, bold values indicate statistically significant differences

^b^compared with nonparametric Mann–Whitney test

### Measures of obesity

The mean post-treatment measures of obesity are presented in Table 1. At the end of the study, weight and BMI were significantly decreased in both groups (all p-values <0.01). Subjects treated with COMBO lost on average 3.6 ± 2.5 kg compared with a 6.3 ± 3.7 kg weight loss in LIRA3 group (*p =* 0.062, Table 2). BMI decreased for 1.3 ± 0.9 kg/m^2^ in COMBO arm compared to 2.2 ± 1.3 kg/m^2^ in LIRA arm with the statistically significant between treatment differences in BMI (*p =* 0.05). LIRA3 intervention also resulted in a significant reduction of waist circumference. The between treatment difference in the decrease of waist circumference was statistically significant (Table 2). Clinically meaningful ≥5% weight reduction was achieved in 35.7% of patients in COMBO and 57.1% of patients in LIRA3 arm (OR = 0.42, 95% CI =0.09–1.91), however the between treatment difference was not statistically significant.

**Table 2:** Comparison of absolute change in clinical parameters of PCOS patients among different treatment groups

	LIRA3	LIRA1.2 + MET2000	*P* ^a^
Characteristic	Absolute change mean ± SD	Absolute change mean ± SD	
Weight (kg)	−6.3 ± 3.7	−3.6 ± 2.5	0.062
BMI (kg/m^2^)	−2.2 ± 1.3	−1.3 ± 0.9	**0.050**
Waist circumference (cm)	−4.2 ± 3.4	−2.2 ± 6.2	**0.014**
Androstenedione (nmol/l)	0.6 ± 2.7	−2.0 ± 6.0	0.376
Total testosterone (nmol/l)	−0.2 ± 0.3	−0.3 ± 0.6	0.285
Free testosterone (pmol/l)	0.6 ± 3.3	−0.7 ± 5.3	0.482
SHBG (nmol/l)	7.3 ± 14.2	19.5 ± 71.4	0.376
Glu 0 min OGTT (mmol/l)	−0.1 ± 0.4	−0.4 ± 0.6	0.734
Glu 120 min OGTT (mmol/l)	−1.2 ± 0.8	−1.1 ± 1.4	0.804
Insulin 0 min OGTT (mU/l)	−2.5 ± 9.3	−7.7 ± 16.3	0.571
Insulin 120 min OGTT (mU/l)	−30.9 ± 29.5 [2]	−9.7 ± 73.4	0.560
HOMA IR	−0.7 ± 2.3	−2.1 ± 3.9	0.427
Cholesterol (mmol/l)	0.0 ± 0.4	−0.3 ± 0.6 [1]	0.094
HDL cholesterol (mmol/l)	0.0 ± 0.1	0.0 ± 0.1 [1]	0.793
LDL cholesterol (mmol/l)	0.0. ± 0.3	−0.3 ± 0.4 [1]	**0.038**
TG (mmol/l)	0.1 ± 0.4	−0.2 ± 0.4 [1]	0.128

^a^compared with nonparametric Mann–Whitney test, bold values indicate statistically significant differences

### Metabolic parameters

Significant decreases of glucose at 0 and 120 min, insulin at 0 min of OGTT, HOMA-IR and LDL cholesterol were observed in the COMBO arm (*p =* 0.015, *p =* 0.016, *p =* 0,035, *p =* 0.013 and *p =* 0.049 respectively, Table 1). LIRA3 resulted in significant improvement of glucose and insulin levels at 120 min of OGTT (*p =* 0.002 and *p =* 0,008 respectively, Table 1). The between treatment difference in the parameters of glucose homoeostasis were not statistically significant, whereas COMBO was superior in the decrease of LDL cholesterol (Table 2).

### Endocrine parameters

Significant decrease of total testosterone was observed in COMBO arm (*p =* 0.023), whereas the decrease in LIRA3 was not statistically significant yet. Androstendione and free testosterone decreases tended to be greater in COMBO compared to LIRA3. In LIRA3 significant increase of SHBG (*p =* 0.018) was observed (Table 1). There were no significant between treatment differences in endocrine parameters (Table 2).

The most common side effects in LIRA3 were nausea (8/14) and diarrhea (5/14). Vomiting occurred in 1/14 patients and 2/14 mild headache were documented. Most of the side effects were present in the first 4 weeks of the treatment, from the 4th to 8th week 2 women had persistent mild nausea. Adverse effects reported in COMBO were nausea (6/14), mild diarrhea (6/14), and insomnia (1/14). All adverse events resolved within the first 1 to 4 weeks. Hypoglycemic event was reported once in 1 woman in LIRA3. No side effect was documented by 6/14 in LIRA3 arm and 8/14 in COMBO arm. No subject withdrew because of the adverse events in either group. One woman from LIRA3 had gallbladder related symptoms one week after the end of the study.

## Discussion and conclusions

Short-term treatment with high dose liraglutide 3 mg monotherapy or liraglutide 1.2 mg in adjunct to metformin both resulted in significant within treatment weight and BMI decrease in obese PCOS. Liraglutide 3 mg was linked to greater weight reduction and higher proportion of good responders that lost at least 5% of baseline weight within 12 weeks. In addition, liraglutide 3 mg led to significant reduction in waist circumference. However, a dual-targeting combination treatment with low dose liraglutide 1.2 mg and metformin resulted in improved androgen profile that was not observed with LIRA3. Both regimens improved parameters of glucose homeostasis. In addition, COMBO led to significant within treatment LDL decrease. Liraglutide 1.2 mg in adjunct to metformin and liraglutide 3 mg as a monotherapy were both generally well tolerated with nausea being more frequent yet transient with liraglutide 3 mg.

Clearly, obesity and obesity related abnormalities are multifactorial diseases. Treatment algorithms for weight management should therefore be tailored to specific obesity related population. In obese PCOS the treatment should focus on modifiable weight related and weight nonrelated derangements. The combination treatment could potentially improve treatment outcomes in obese PCOS via co-targeting multifactorial origin of obesity and concomitant abnormalities intrinsically related to PCOS. Furthermore, the right composition of combination treatment could enhance the therapeutic index of the drugs in combination.

Metformin as an add-on to low dose liraglutide seems to be mechanistically well-suited combination when obesity is related to PCOS. Metformin alone has well known beneficial metabolic and gynecological effects in this population that are mediated through improvement of IR and via direct action at the ovarian level beyond weight reduction [13]. Similarly, adding metformin to liraglutide could potentially enhance GLP-1 effect of liraglutide enabling the use of lower dose of liraglutide in combination treatment. It was demonstrating in animal studies that metformin increased GLP-1 [22]. In humans a single dose of metformin also increases GLP-1 after oral glucose load in non-diabetic subjects [21]. In addition, chronic exposure to metformin increased GLP-1 in diabetic and non-diabetic participants [21]. The impact of metformin on weight reducing potential of liraglutide could be further explained also through the stimulation of the expression of GLP-1 receptor and insulin induced signalling pathways [19].

We have previously demonstrated that low dose liraglutide 1.2 mg in combination with metformin in obese patients with PCOS who had lost less than 5% of body weight with metformin and lifestyle intervention had beneficial effects compared to low dose liraglutide and metformin monotherapies. After randomization the participants either continued with metformin or were switched to liraglutide 1.2 mg alone or assigned liraglutide 1.2 mg in combination with metformin for 12 weeks. Combination led to 6.5 kg loss compared with 3.8 loss in low dose liraglutide arm and 1.2 kg in metformin arm [26]. Furthermore, we observed that metformin added to low dose liraglutide 1.2 mg was more effective than liraglutide 1.2 mg alone in reducing weight after 12 weeks in treatment naïve obese PCOS. Addition of metformin resulting in higher proportion of subjects achieving clinically meaningful ≥ 5% weight reduction in almost 60% compared to about 40% of good responders in low dose liraglutide monotherapy arm [27]. Both study designs were conducted with low dose liraglutide 1.2 mg before high dose liraglutide 3 mg was established as an anti-obesity treatment.

The present study further supports liraglutide 3 mg for weight reduction in obese PCOS. In 12 weeks almost 60% of women exposed to liraglutide 3 mg achieved clinically meaningful ≥ 5% weight loss, that might already improved cardiovascular risk profile in obese patients [28]. In addition, high dose liraglutide led to a significant within treatment reduction of waist circumference, another well-known independent risk factor for cardiovascular disease. In line with our observation, dual-energy x-ray absorbtiometry of obese patients who were exposed to liraglutide 3 mg for 20 weeks demonstrated that weight reduction was primarily from decreased fat, including visceral fat, rather than lean tissue [29]. However, a significant weight reduction in LIRA 3 group was not yet associated with improvement of androgen profile.

The combination treatment of liraglutide 1.2 mg and metformin also resulted in significant within treatment reduction of weight in obese PCOS, although the amount of weight and waist circumference reductions and the proportion of good responders that lost ≥ 5% baseline weight were less than in LIRA3 arm. However, a dual-targeting treatment approach was superior in improving the androgen profile. The observed superior endocrine effect with COMBO despite the inferior trend in weight reduction could imply well-known weight independent action of metformin mediated through direct regulation of ovarian steroidogenesis [13].

The beneficial effects on glucose metabolism were comparable in both treatment arms, whereas the LDL decrease in our study was greater on COMBO compared to LIRA3. Previous studies with liraglutide have shown no consistent changes in lipids in subjects with type 2 diabetes [9]. Due to small sample size, general methodological difficulties in androgen measurements we cannot provide any firm conclusion about observations regarding endocrine and metabolic parameters that were pre-specified as secondary outcomes.

High dose liraglutide was not related to deterioration in general tolerability except higher frequency of dose dependent nausea. It was present in almost 60% of women treated with liraglutide 3 mg compared to less than half of women receiving COMBO. However, all side effects were transient and mild to moderate, declining within 4–6 weeks. The adverse events were in line with the documentation of dose depending adverse events in phase 2 trial with liraglutide from 1.2 mg up to 3.0 mg, where 3.0 mg dose induced nausea in almost 50% of participants without type 2 diabetes [30]. In phase 3 trials nausea was induced with lower doses 1.2 and 1.8 mg in up to 40% of diabetic individuals [31].

The results of our study are subject to several limitations. The duration was too short to assess the sustainability of the benefits from the combination regimen. Our sample size was small. Future larger designs of longer duration including the proposed combination treatment in PCOS related obesity could possibly be powered using our preliminary results. The main strength of this study was pathophysiological supported strategy combining two medications that act through synergistic modulation of incretin axis and via direct and indirect manipulation of intrinsic weight independent abnormalities in this specific obesity related population.

Overall, the selection of the right combination for the combination treatment is a complex task that requires understanding of pathophysiological backgrounds of the obesity and obesity related comorbidities. The results of this study indicated the potential benefits of combination of low dose liraglutide and metformin in the management of obese PCOS. Our results are preliminary, but support future investigations of this dual treatment approach when obesity is linked to PCOS.

## Abbreviations

BMI: Body mass index
COMBO: Combination of metformin and liraglutide
GLP-1: Glucagon-like peptide-1
HOMA-IR: Homeostasis model assessment of insulin resistance
IR: Insulin resistance
LIRA: Liraglutide
MET: Metformin
OGTT: Oral glucose tolerance test
PCOS: Polycystic ovary syndrome
SD: Standard deviation
SHBG: Sex hormone binding globulin
